# Long-term, unilateral third cranial nerve palsy, ocular myositis, and high CSF interleukine-2 persisting for 14 months after mild SARS-CoV-2 infection-case report

**DOI:** 10.1016/j.clinsp.2024.100331

**Published:** 2024-02-06

**Authors:** Carla A. Scorza, Josef Finsterer, Fulvio A. Scorza

**Affiliations:** a Disciplina de Neurociência, Universidade Federal de São Paulo/Escola Paulista de Medicina (UNIFESP/EPM). São Paulo, SP, Brazil; b Neurology & Neurophysiology Center, Vienna, Austria

## Commentary

SARS-CoV-2 Infections (SC2I) are known to manifest not only in the lungs but also in extrapulmonary organs, particularly the central and Peripheral Nervous System (CNS, PNS).^1^ One of the PNS manifestations is radiculitis, which can affect not only peripheral but also cranial nerves.^2^ The cranial nerve most commonly affected in SC2I is the facial nerve, but all other cranial nerves can also be affected unilaterally or bilaterally.^2^ Third Cranial Nerve Palsy (3rdCNP) in SC2I is rare^3^, ^4^, ^5^ and myositis of the extraocular muscles is even rarer.^6^^,^^7^ Here the authors report a unique patient with long-term, unilateral 3rdCNP four weeks after mild SC2I.

**Fig. 1:**
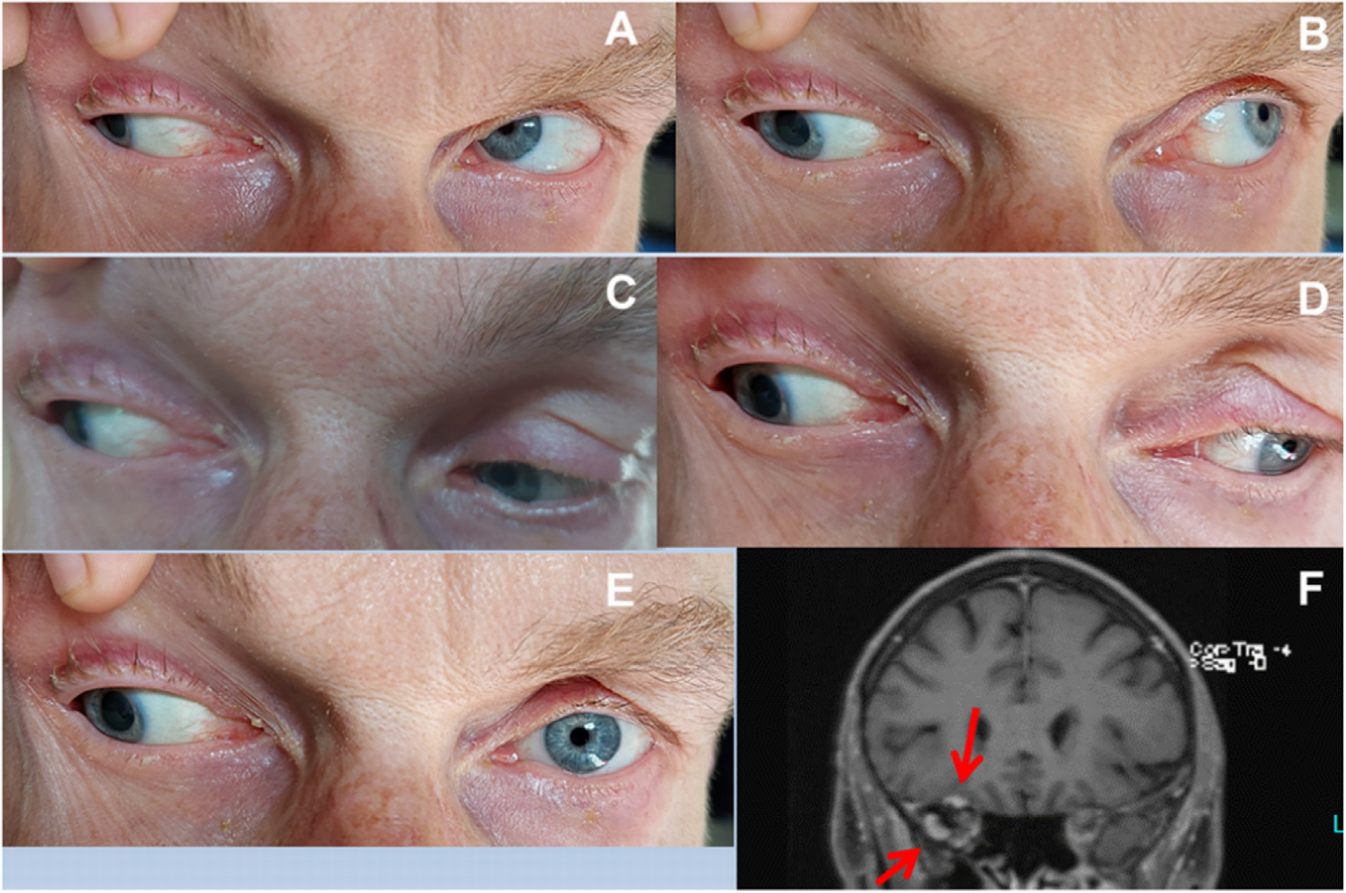
Right third cranial nerve palsy in the index patient (A: Gaze to the top right; B: Gaze to the top left; C: Gaze to the bottom right; D: Gaze to the bottom left, E: Gaze straight forward, F: Coronary T1-weighted image with contrast showing enhancement of the right superior lateral rectus muscles).

The patient is a 52-year-old male who developed incomplete 3rdCNP in August 2022, which progressed until November 2022. Since then, the palsy has remained unchanged. Four weeks before the onset of 3rdCNP, the patient tested positive for SARS-CoV-2 and presented with mild COVID-19 (WHO classification), which resolved within 4 days. 3rdCNP manifested by ptosis, fixed abduction, and downward positioning of the right globe when looking in any direction, and dilatation of the right pupil (Fig. 1). The remainder of the clinical neurological exam was normal. The erythrocyte sedimentation rate was 30 mm after one hour. Thyroid function, ANA, ANCA, antibodies to Borrelia and the virus panel were negative. Cerebrospinal Fluid (CSF) studies revealed elevated proteins and markedly elevated interleukin-2 (n, 4750 pg/mL <3000pg/mL). Cerebral MRI showed enhancement of the superior rectus and lateral rectus muscles (Fig. 1). FDG-Positron Emission Tomography (FDG-PET) showed hypermetabolism of these muscles.

Although the patient was treated with prednisone (70 mg/day) since March 2023, which was continuously tapered to 5 mg/day, there was little improvement after 12 and 14 months. He did not receive Intravenous Immunoglobulins (IVIG), Plasma Exchange (PLEX), or Rituximab (RTX). Alternative causes of 3rdCNP that were ruled out included intracerebral nuclear lesions due to vascular disease, demyelination, neoplasms, diabetes, aneurysm, or extradural hematoma involving the basilar portion of the nerve, lesions of the intra-cavernous portion (pituitary apoplexy, carotid-cavernous fistula), lesions of the intraorbital portion (neoplasm, trauma, Tolosa-Hunt syndrome), and muscle diseases such as myositis, vasculitis, myasthenia, or thyroid orbitopathy.

The patient presented is of interest for unilateral 3rdCNP that had been present for 14 months following a mild SARS-CoV-2 infection four weeks before the onset of the palsy. Arguments for a causal relationship are that alternative causes for 3rdCNP have been thoroughly ruled out, that 3rdCNP after SC2I has been previously reported,^3^, ^4^, ^5^ and that interleukine-2 was significantly elevated in the CSF. Increases in interleukine-2 have been repeatedly reported in patients with CNS/PNS involvement of SC2I.^8^^,^^9^ Another argument for a causal relationship between COVID-19 and 3rdCNP is that 3rdCNP has also been reported after vaccination against SARS-CoV-2.

Whether the enhancement of the right superior and lateral rectus muscles represents immune-mediated ocular myositis, fibrosis, or is just a secondary defect due to long-term denervation remains speculative, as there are arguments for and against ocular myositis. Arguments in favor of ocular myositis are that it has been previously reported as a complication of SC2I,^6^^,^^7^ that there was no abducens nerve palsy that could explain T2-hyperintensity of the lateral rectus muscle, and that other ocular muscles, also innervated by the oculomotor nerve, showed no enhancement. Another strong argument for myositis is that the two muscles showed hypermetabolism on FDG-PET. Denervation or fibrosis would be associated with hypometabolism rather than hypermetabolism. Arguments against ocular myositis are that the patient did not complain about spontaneous pain or pain on eye movements, that only two ocular muscles were enhanced, and that long-term denervation of the extra-ocular muscles can lead to atrophy and T2-hyperintensity.^10^

The pathophysiology of 3rdCNP remains speculative, but it can be assumed that it evolved in response to the humoral or cellular immune response that was not only directed against the virus but also cross-reacted with axons or myelin sheaths of this nerve. Why this specific nerve responded with such a misdirected immune response remains speculative, but previous subclinical axonal or myelin damage may have favored the development of 3rdCNP. However, there was no evidence of prediabetes, chronic renal failure, chronic toxin exposure, vitamin deficiency, a history of chemotherapy, or chronic alcoholism.

This case is the first to demonstrate that SC2I can be complicated by long-term, isolated, unilateral 3rdCNP and ocular myositis, that the palsy and ocular myositis can be unresponsive to steroids, and that 3rdCNP can persist for years. Ophthalmologists and neurologists should be aware of such SC2I complications and immediately take all necessary diagnostic and therapeutic measures to improve the outcome of these patients.

## Ethics approval

Was in accordance with ethical guidelines. The study was approved by the institutional review board.

## Consent to participate

Was obtained from the patient's parents.

## Consent for publication

Was obtained from the patient's parents.

## Availability of data

All data are available from the corresponding author.

## Code availability

Not applicable.

## Authors’ contributions

JF: Design, literature search, discussion, first draft, critical comments, final approval, CS and FS: Literature search, discussion, critical comments, final approval.

## Funding

No funding was received.

## Conflicts of interest

The authors declare no conflicts of interest.
